# Multiscale description of avian migration: from chemical compass to behaviour modeling

**DOI:** 10.1038/srep36709

**Published:** 2016-11-10

**Authors:** J. Boiden Pedersen, Claus Nielsen, Ilia A. Solov’yov

**Affiliations:** 1 Department of Physics, Chemistry and Pharmacy, University of Southern Denmark, DK-5230 Odense M, Denmark

## Abstract

Despite decades of research the puzzle of the magnetic sense of migratory songbirds has still not been unveiled. Although the problem really needs a multiscale description, most of the individual research efforts were focused on single scale investigations. Here we seek to establish a multiscale link between some of the scales involved, and in particular construct a bridge between electron spin dynamics and migratory bird behaviour. In order to do that, we first consider a model cyclic reaction scheme that could form the basis of the avian magnetic compass. This reaction features a fast spin-dependent process which leads to an unusually precise compass. We then propose how the reaction could be realized in a realistic molecular environment, and argue that it is consistent with the known facts about avian magnetoreception. Finally we show how the microscopic dynamics of spins could possibly be interpreted by a migrating bird and used for the navigational purpose.

Numerous birds travel large distances every year when migrating across the continents, utilizing various cues to find their way. A particularly remarkable cue is the magnetic field of the Earth, whose direction and intensity the birds are able to detect and use for navigation^1^. This geomagnetic field has a strength of about 50 *μ*T, and the detection of such weak magnetic fields by a biological system is a difficult task. In this investigation we are dealing with one of the mechanisms that may explain this exciting phenomenon, in particular the mechanism that relies on the quantum spin dynamics of transient photoinduced radical pairs^2^,^3^,^4^,^5^,^6^,^7^,^8^,^9^,^10^,^11^,^12^,^13^,^14^,^15^, originally suggested by Schulten *et al*. in 1978^5^ as the basis of the avian magnetic compass sensor.

The radical pair mechanism of the avian magnetic compass deals with the quantum evolution of highly non-equilibrium electron spin states of pairs of transient spin-correlated radicals residing inside a bird’s retina as featured in Fig. 1. These spin-correlated radicals could form electronically entangled singlet and triplet states, which are respectively characterized by an anti-parallel and parallel alignment of the unpaired electron spins of the radicals. The core of the radical pair mechanism of avian magnetoreception relies on the possible engagement of the radicals in biochemical reactions, that could be affected by magnetic fields even though the Zeeman interaction of an unpaired electron spin with the geomagnetic field is more than six orders of magnitude smaller than the thermal energy available inside the ‘wet, warm and noisy’ biological surroundings. Hence from a classical perspective, a magnetic sensitivity should never arise in biochemical reactions, and we must, therefore, rely on the intervention from quantum effects. Such quantum effects enters the stage through the radical pair mechanism, which so far is the only known way an external magnetic field can influence a chemical reaction^1^,^9^,^16^,^17^,^18^,^19^. The radical pair mechanism has been studied for about half a century by now, and has been successfully applied to various phenomena such as spin polarizations^20^ and magnetic isotope effects^21^.

In order to act as a reliable chemial compass a radical pair reaction must satisfy a number of conditions, which have been extensively discussed in earlier publications^1^,^2^,^9^, and a recent review in particular^1^. These conditions involve chemical, magnetic, kinetic, structural and dynamical properties of a radical pair, and the key point of the radical pair mechanism is the spin selectivity of a chemical reaction. Previous spin chemical models for the avian magnetic compass have either suggested that different reaction products are formed from the different spin-correlated states of the underlying radical pair^6^, i.e. different singlet and triplet reaction products are expected to emerge and in turn trigger different neurological responses in bird behaviour, or have assumed a model similar to the one presented here^2^,^22^, but without investigating the benefits of a *fast* spin-dependent regeneration reaction. In both models, the relative yields of the different reaction pathways could be manipulated by reorientation of the radical pair in the magnetic field: the anisotropy of the internal magnetic interactions in the radical pair, i.e. the hyperfine interactions, define a molecular coordinate system, that in turn determines the orientation between the radical pair and the magnetic field as illustrated in Fig. 1 using just a single angle Θ.

The present study seeks to establish a multiscale link between the radical pair model of the chemical compass and bird behaviour. For this purpose we introduce a generic model for the avian chemical compass, and then link it to several established facts about the magnetic sense in birds. The model describes a possible cyclic reaction scheme with radical pair intermediates. Cyclicity of radical pair reactions has only received little attention previously in connection to the chemical compass of birds^23^, while it apparently leads to a surprisingly precise navigational device, as compared to most of those proposed earlier. The main feature of the proposed reaction scheme is a fast spin-dependent regeneration reaction, which facilitates the regeneration of the initial molecular system that hosts the magnetically sensitive radical pair. Based on the model reaction scheme, we suggest some possibilities for realising this model in a realistic biological environment. Finally we investigate the implications of the microscopic chemical compass model on the macroscopic scale, and demonstrate the navigational capabilities of birds relying purely on the magnetic compass. The present investigation attempts to bridge together the various scales: electronic, molecular and organism in the truly multidisciplinary problem of avian magnetoreception. Historically these three scales were treated separately, while we seek to fill in some gaps between them.

## The spin compass model

The core of the proposed spin compass relies on two irreversibly connected radical pairs (RPs), RP1 and RP2, with different magnetic interactions and a *fast* spin-dependent regeneration reaction originating from the primary RP1 as shown in Fig. 2. This fast regeneration reaction is an essential ingredient of the suggested model – with emphasis on *fast*. Similar models have been successfully used in other biochemical systems^24^,^25^,^26^,^27^ but have not been applied to the spin compass of birds. A regeneration reaction that provides magnetic field selectivity of the radical pair reactions has been postulated in some of the earlier similar investigations as the so-called back reaction or recombination reaction^2^,^28^,^29^,^30^, but here it is reviewed from a different chemical and physical perspective, particularly, the possibility of a **fast** regeneration reaction has not been explored.

The generic radical pair model considered in the present investigation is illustrated in Fig. 2. Pairs of radicals are created instantaneously, after irradiation of a photoreceptor molecule P by light and subsequent electron transfer. The primary radical pair, RP1, forms a singlet electron spin state, but due to magnetic interactions it has the possibility to transform to the triplet electron spin state.

The primary radical pair, RP1, reacts irreversibly to produce a secondary radical pair, RP2, with conservation of the spin state. The secondary RP2 undergoes some chemical transformation to form the reaction product E. Creation of the product state E is assumed spin dependent in this model, i.e. only singlet radical pairs are allowed to react. The dominant interaction in the secondary RP2 is spin relaxation which will make the distribution of singlet and triplet states approach equilibrium. Thus, when singlet pairs react the relaxation will cause some triplet pairs to be transformed into singlet pairs. Therefore, all secondary RP2s will eventually decay through the singlet channel and thus end up in the same product state E. Note that the singlet-only reaction of RP2 and associated spin relaxation is a convenient but not necessary assumption. The following two alternatives give the same results: for a fast regeneration reaction, practically all RP2 will be created in a triplet state and thus if the triplet RP2 state reacts to form E, then all RP2 will end up in E, and spin relaxation is not needed. Even more straight forward, if the reaction of RP2 to form E is spin independent, then clearly all RP2 states again will end up in E.

The chemical compass of a bird is assumed to operate by changing the rate of formation of the product state E, or any later derivative of E, while the product state is cyclically converted into the initial photoreceptor molecule P. The reaction cyclicity is crucial for the studied mechanism, as it leads to a significant enhancement of the compass sensitivity in respect to magnetic field direction. Thus, for example, a significant enhancement of the magnetic field effect for a cyclic reaction scheme has been reported in a recent study by Kattnig *et al*.^23^.

Obviously, no magnetic field effect on the formation of the product state E is possible without spin dependent reactions, since all photo-generated radical pairs, RP1, would end up in the same product state. Therefore, a crucial point is the introduction of a fast spin dependent regeneration reaction that allows the primary singlet radical pair, RP1, to recombine and produce the original photoreceptor molecule, either directly or by a series of reactions. The crucial condition, that will be explored in the following, is that the rate constant of the regeneration reaction, *k*_*r*_, is significantly larger than the rate constant for formation of RP2, *k*_*f*_, i.e. the condition *k*_*r*_ ≫ *k*_*f*_ is expected to hold. This condition effectively means that the magnetic field sensitivity of RP1 will be multiplied during the many photoexcitation-and-regeneration-reaction cycles, before the system finally ends up in the signalling state. In other words, the “loop” shown by the curved green arrow in Fig. 2 basically functions as a signal amplifier. This can be illustrated more specifically if one defines 0 ≤ *p*_*f*_ ≤ 1 as the probability that the system acquires the signaling state after a photoactivation, and *p*_*r*_ = 1 − *p*_*f*_ as the probability of the system to take the regeneration reaction pathway instead. Introducing *λ* as the number of consecutive times the magnetoreceptor is excited, the probability that the system acquires the signaling state is:


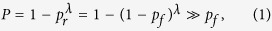


which demonstrates that once *p*_*f*_ and *p*_*r*_ change, for example due to magnetic fields, the effect will be enhanced substantially. The number of excitation cycles, *λ*, that would be needed to obtain an observable signal, would depend on both rate constants, *k*_*r*_ and *k*_*f*_, and the singlet to triplet interconversion rate, *k*_*S*↔*T*_.

The mechanism explored here has many possible biological realisations, as it basically relies on two different reaction pathways occuring within a magnetoreceptor molecule: a spin dependent and a spin independent one, where the spin dependent reaction is expected to occur much faster.

## Methods

The model employed in the present investigation has been discussed earlier^26^, but never in the context of a chemical compass of birds. For the sake of completeness, the basic physics of the model is reviewed in this section, while the next section reveals the novel aspects of the chemical compass that could be learned from it.

### Angular dependence of the product rate of formation

To illustrate how the present model of a chemical compass renders a precise navigational device, we use the same spin Hamiltonian for the radical pair, RP1, as Ritz *et al*.^6^:


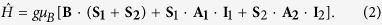


**Figure 1:**
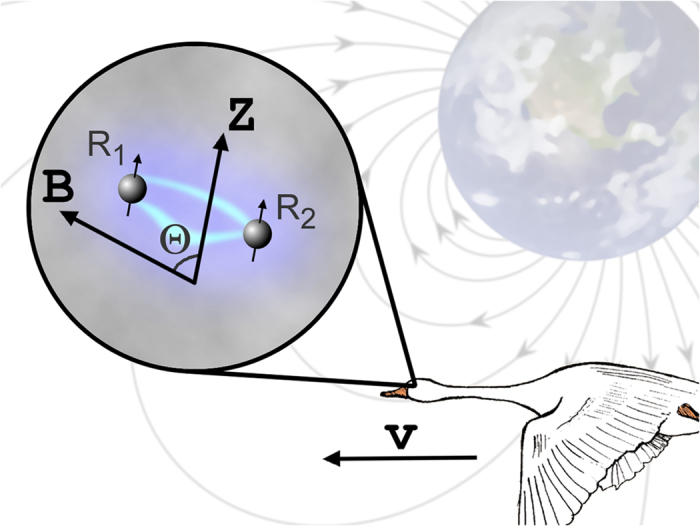
Schematic illustration of the avian radical pair-based compass. Magnetoreceptive molecules in the birds eyes host a pair of radicals (*R*_1_, *R*_2_) and endow the bird with capabilities to sense the Earth’s magnetic field. In the most simplified case, each radical pair is associated with a coordinate frame such that internal magnetic interactions are considered isotropic in the *xy*-plane, while the anisotropy defines the *z*-axis. The radical pairs participate in spin-dependent chemical reactions that are sensitive to the angle Θ between this z-axis and the direction of the geomagnetic field *B*, which in turn could be related to the direction of bird motion, denoted by *v*.

Here the first term is the Zeeman interaction where *μ*_B_ is the Bohr magneton, *g* = 2 is the assumed identical isotropic g-values of the radicals, **B** = *B*_0_(sin Θ; 0; cos Θ) is the geomagnetic field with an absolute value of *B*_0_ = 50 *μ*T and direction described through the angle Θ, introduced in Fig. 1. **S**_**1**_ and **S**_**2**_ are the spin operators of the unpaired electronic spins in the two radicals. The second and the third terms in Eq. (2) are the hyperfine interactions arising in the two radicals; for simplicity we consider both radicals as having a single nucleus of spin-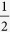, in addition to the single unpaired electron. The first nucleus is assigned an axially symmetric hyperfine tensor **A**_**1**_ which is diagonal in the *x*, *y*, *z*-coordinate system of RP1 with the values (1, 1, 0) mT, while the second nucleus is considered to have an isotropic hyperfine tensor, **A**_**2**_, with the diagonal values of (0.5, 0.5, 0.5) mT.

In principle, exchange and dipole-dipole interactions should also be included in the spin Hamiltonian, Eq. (2). These interactions generally destroy the compass sensitivity, however, under some circumstances could become negligible, or even cancel each other^31^. Since we consider a model that serves as an illustration for the avian chemical compass, we will assume that this is indeed the case, and will further on neglect the exchange and dipole-dipole interactions.

**Figure 2:**
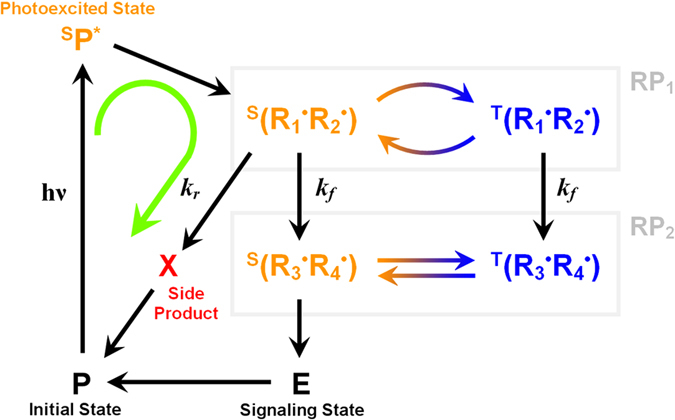
Radical pair reaction scheme with fast regeneration reaction. The radical pair RP1 is formed in the primary magnetoreceptor, P, immediately after its photoexcitation. The initially singlet radical pair 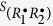 converts to the triplet form 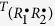 due to the presence of anisotropic hyperfine interactions in the radicals and the geomagnetic field. RP1 could be converted to the secondary radical pair RP2 through a spin independent reaction; the conversion occurs with the rate constant *k*_*f*_. Alternatively, the singlet radical pair 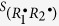 is subject to a fast regeneration reaction that occurs with a rate constant *k*_*r*_, such that *k*_*r*_ ≫ *k*_*f*_, leading to a biologically inactive side product state of the primary receptor. Because the regeneration reaction is assumed to be fast, many cycles of side product formation are expected to happen (following the green curved arrow) before any significant amount of RP2 is obtained, which is thought to be a crucial intermediate state of the magnetoreceptor prior to its conversion to the biological signalling state, E. The side product and the radical pair RP2 states of the receptor transform further and eventually regenerate to the initial state of the receptor.

The spin dynamics of the primary radical pair, RP1, requires solving the stochastic Liouville equation, the equation of motion for the spin density matrix, *ρ*, of the radical pair^32^. The rate equation for the spin density matrix of the primary RP1 can be written as^26^:


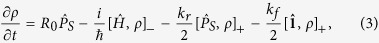


where 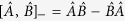 and  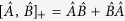 denote the commutator and anti-commutator, respectively, and **1** is the identity matrix. The first term on the right hand side of Eq. (3) represents the rate of formation of RP1 in the singlet electron spin state; 
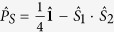
 is the singlet electron spin projection operator, and *R*_0_ is the actual rate of formation of the initial RP1 after irradiation of the host molecule P. The second term describes the coherent quantum mechanical evolution of the spin state of RP1. The third term describes the spin selective regeneration reaction of RP1, which is described by the anti-commutator form^26^,^33^,^34^, where *k*_*r*_ is the regeneration rate constant of the singlet RP1. The last term describes the chemical reaction that transforms the primary radical pair into the secondary RP2 through the forward reaction with rate constant *k*_*f*_. The density matrix, *ρ*, is the key measure of the chemical compass as it contains information about all reactions involving RP1 included in the scheme in Fig. 2. When enough light is available to continuously excite the initially inactive primary receptor, the radical pair RP1 is formed at a rate, *R*_0_, in the singlet state. Due to the cyclicity of the reaction scheme, one expects the density matrix, *ρ*, to reach a steady state, i.e. the density matrix describing the time evolution of the spin system becomes constant in time, being governed by the following condition^26^:


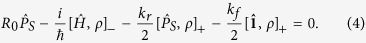


The rate of formation of the secondary radical pair, *R*_2_, can thus be readily computed once the density matrix of the primary radical pair is known, as


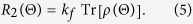


Here the density matrix, *ρ* ≡ *ρ*(Θ), is the solution of Eq. (4). Note, that the density matrix and thus the rate of formation of the secondary RP2 is proportional to *R*_0_, and thus it is convenient to express the rate of formation of RP2 in dimensionless units as


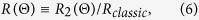


where


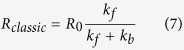


Is the classical rate of product formation, i.e. the rate in the absence of quantum mixing. Note that *k*_*f*_/(*k*_*f*_ + *k*_*b*_) is the fraction of generated RPs that gives rise to the product E and thus leads to a signaling state, in the absence of mixing. The reciprocal is therefore the minimum number of photons needed to get one signaling state. The dimensionless quantity *R*(Θ) in Eq. (6) indicates the effective number of spin conversion channels opened by the magnetic interactions^35^ and it is, therefore, restricted by the actual interactions experienced by the radical pair.

The rate of RP2 formation could be decomposed into the rate of RP2 singlet, *R*_*S*_, and triplet, *R*_*T*_, states formation which are useful for comparison with earlier studies and are defined as:


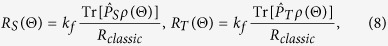


where


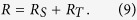


Since in the postulated model the secondary radical pair, RP2, is linked with formation of the product state E, see Fig. 2, the rate *R*(Θ) in Eq. (6) also defines the rate of product formation, which is discussed in the following.

## Results and Discussion

Employing the postulated model of a chemical compass, we explore its properties through analyzing the compass precision that depends on the choice of the rate constants, *k*_*r*_ and *k*_*f*_.

### Rate of product formation

**Figure 3:**
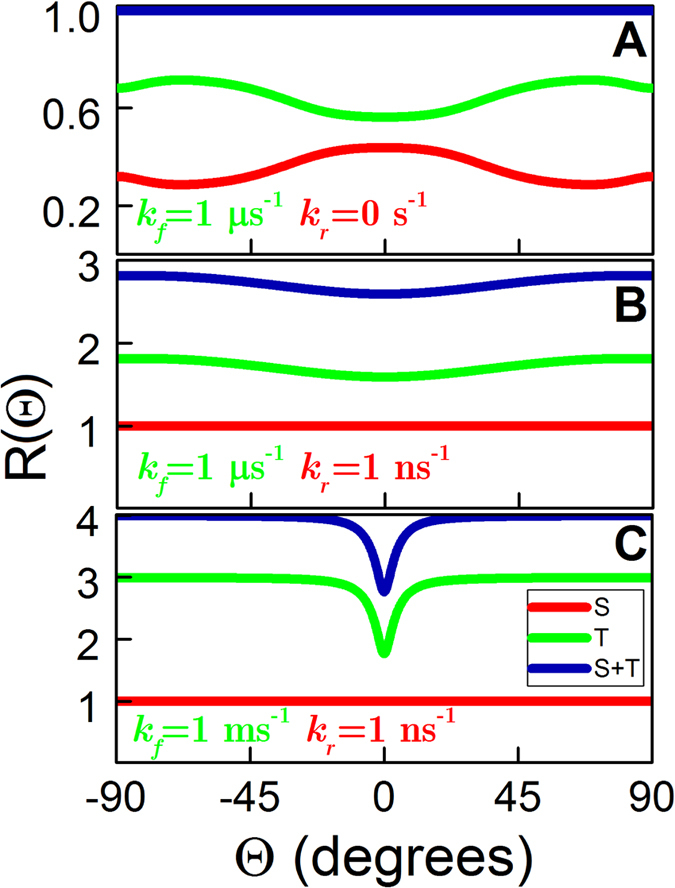
Product formation rates for the chemical compass model. Dimensionless product formation rates, defined in Eqs (6) and (8), of the radical pair reaction introduced in Fig. 2, as a function of the radical pair orientation with respect to the geomagnetic field, see Fig. 1. The considered examples correspond to a model without a regeneration reaction (**A**), consistent with an earlier study^6^, and to a model with a fast regeneration rate constant *k*_*r*_ = 1 ns^−1^, (**B**,**C**). In the latter cases the value of the forward rate constant *k*_*f*_ is taken different. Color indicates *R*_*S*_ (S, red), *R*_*T*_ (T, green) and the total rate of product formation *R* (S + T, blue).

Figure 3 illustrates the orientational dependency of *R*(Θ), defined in Eq. (6), calculated for the reaction scheme depicted in Fig. 2 with and without the regeneration reaction. Without a regeneration reaction (*k*_*r*_ = 0 s^−1^), *R*(Θ) is always unity as shown in Fig. 3A. In order to have any angular dependence of the product formation rate in this case, one must consider only the rate of formation from one of the correlated spin states, e.g. *R*_*T*_(Θ) or *R*_*S*_(Θ) as used in ref. 6. The results shown in Fig. 3A were reported earlier^6^, but are included to illustrate the fundamental differences in the orientational dependence between the previous results, and those derived from the present model, Fig. 3B,C.

The model with a regeneration reaction has a different behaviour, see Fig. 3B,C. In these cases *R*_*S*_(Θ) is unity and thus independent of the magnetic field direction. The angular dependence originates from *R*_*T*_(Θ) and should, according to the theory^35^, be smaller than or equal to the number of channels opened by the magnetic interactions in the radical pair. The maximal value of *R*_*T*_(Θ) in the case of *k*_*f*_ = 1 *μ*s^−1^, *k*_*r*_ = 1 ns^−1^ (Fig. 3B) is 1.8, while the values of *R*_*T*_(Θ) are higher for *k*_*f*_ = 1 ms^−1^, *k*_*r*_ = 1 ns^−1^, as demonstrated in Fig. 3C.

In order to obtain a precise compass, the forward rate constant must be significantly smaller than the magnetic field dependent singlet-to-triplet conversion rate, which for a magnetic field of 50 *μ*T is of the order *k*_*S*↔*T*_ ≈ 1 *μ*s^−1^, i.e. we require that *k*_*f*_ ≪ *k*_*S*↔*T*_. The rate constant for the regeneration reaction must satisfy *k*_*r*_ ≫ *k*_*f*_ in order for the regeneration reaction to be fast. This is one of the key assumptions of the proposed model, that will be explored in the following. By using a smaller value for *k*_*f*_ than in Fig. 3B it is possible to obtain a higher rate of product formation as displayed in Fig. 3C, and it is seen that for angles, Θ, significantly differing from 0°, *R*_*T*_ → 3, i.e. approaches the limiting value.

A striking observation in Fig. 3C is that there is no change in the rate of product formation for angular reorientations larger than approximately ±20° and, therefore, if a bird’s chemical compass would read the change of the rate of product formation, it would not be able to distinguish any changes of the magnetic field direction beyond ±20°. For angular orientations |Θ| < 20° there is a strong and steep angular dependence of the product formation rate, and just a small change of direction will cause a large change of product rate of formation. Thus if we assume that the chemical compass dependence on the magnetic field direction is similar to that in Fig. 3C, it is conjectured that the bird essentially can sense only when it moves from one region to the other but that it has no precise sense of the actual product rate and thus the angle.

During adjustment of flight direction the bird essentially moves its head until it senses a signal change which indicates the correct direction. Obviously, the stronger the change the more likely it is that the corresponding direction is chosen as the correct one. This means that the response curve can be seen as a probability density for flight direction determination. In other words, the bird requires periodically to recalibrate the compass and after such readjustment it reads a specific direction where it should go. This result is in a perfect agreement with experimental observations, as birds seem to recalibrate their compass regularly^36^. During calibration, the bird essentially moves its head until it recognizes a signal change which indicates towards a certain direction.

### Chemical compass precision

**Figure 4:**
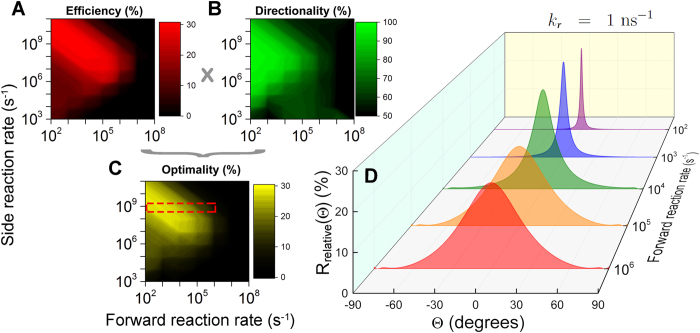
Chemical compass optimality. The radical pair based compass is characterized through the model parameters *efficiency* (**A**), *directionality* (**B**) and *optimality* (**C**), defined in Eqs (11) and (12). The parameters were calculated as function of the forward rate constant *k*_*f*_ and the regeneration rate constant *k*_*r*_, see Fig. 2. Selected dependencies of the relative rate of product formation, Eq. (10), on radical pair orientation for values of *k*_*r*_ ~ 1 ns^−1^ and *k*_*f*_ ~ 10^2^–10^6^ s^−1^ are shown in **D** – these values are highlighted by the red box in **C**. The orientation of the radical pair in the magnetic field is described through the angle Θ, defined in Fig. 1.

It is unlikely that the magnetic compass of birds relies on the absolute rate of product formation, since (i) variation in light intensity will give rise to a larger variation in product yield than changing the orientation of a bird in a magnetic field and (ii) most of the biological signalling pathways read relative information with a much higher precision^37^. Hence, it is conjectured that the bird can only sense *changes* in the product rate of formation. Figure 4D displays a series of relative changes of product formation rates for selected values of the rate constants, *k*_*f*_ and *k*_*r*_. The relative change of the product formation rate is defined as:


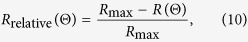


where *R*(Θ) is defined in Eq. (6) and *R*_max_ is the maximal value that *R*(Θ) attains. A relative change of more than 10% is found for all values of the considered forward rate constant, *k*_*f*_. One of the most important observations in Fig. 4D, is that the angular interval where the noticeable change of *R*_relative_ occurs is decreasing with the decrease of the forward rate constant and that in principle any magnitude of standard deviation can be produced. The decrease of the standard deviation of the distributions in Fig. 4D leads to an increase in compass sensitivity, such that the direction could be determined more precisely.

Recently, it has been reported^38^ that a spike-like dependence of the singlet yield of the radical pair reaction could emerge, and was brought in connection with the avian magnetic compass. This effect happened due to an avoided crossing of the energy levels of the RP as a function of its orientation with respect to the magnetic field. The avoided crossing gives rise to maximal mixing of states and thus to a narrow dip (spike) in the singlet yield^39^,^40^, while level crossing corresponds to zero mixing and thus to a narrow increase in the singlet yield^40^. Precisely this effect has been reported for a radical pair system with long lifetime which is, however, different from the effect discussed in the present investigation. On the contrary, the suggested model assumes a short lifetime of the radical pair, which is caused by the fast regeneration reaction. The short lifetime of the radical pair allows to produce an angular dependence of the reaction yield with basically any width by tuning the corresponding rate constant, *k*_*r*_, while an avoided crossing always results in a very narrow spike in the reaction yield. The mechanism proposed in the present investigation, thus, allows for a natural tuning possibility of the compass precision, which may be an important factor during the evolutionary refinement of the compass in birds.

**Figure 5:**
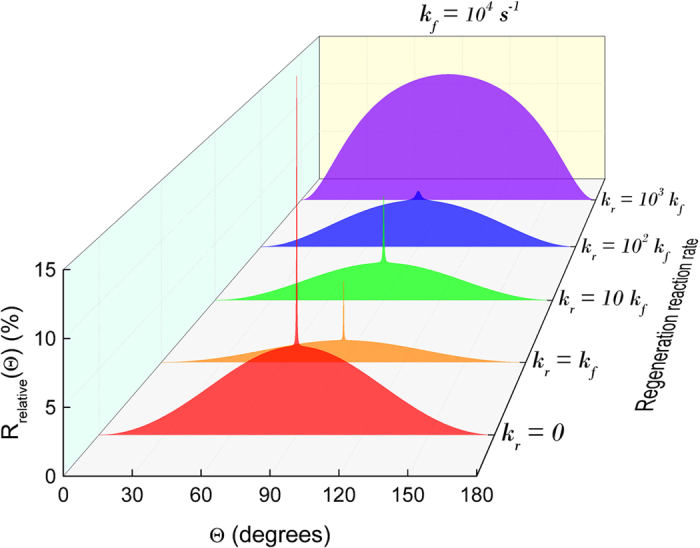
Avoided crossing and regeneration reaction. It has been shown that the narrow spike produced by an avoided crossing can lead to a very precise chemical compass^38^, but as can be seen here, this effect cannot be combined with the effect of having a cyclic reaction scheme with a fast regeneration reaction. In fact these effects are complementary to each other. Note that for *k*_*r*_ = 0, the relative singlet rate, *R*_*S*,relative_(Θ), has been plotted (as in Fig. 3A), whereas for the other calculations the relative total rate, *R*_relative_(Θ), has been plotted (as in Fig. 3B,C). In these calculations the hyperfine tensors are diagonal with the values (−0.0989, −0.0989, 1.7569) mT and (0, 0, 1.0812) mT, chosen consistent with the original publication^38^.

Since two different mechanisms for increased compass precision have now been suggested, it is natural to assume that their combination would operate even better. However, this is not so, as illustrated in Fig. 5, which shows that these mechanisms are complementary to each other. The spike originating from the avoided crossing is incompatible with the increased precision offered by the fast regeneration reaction: note how the curved background of the spike in Fig. 5 changes when increasing the ratio between *k*_*r*_ and *k*_*f*_, especially near Θ = 0° and Θ = 180° where an increasingly steeper change in *R*_relative_ is seen. So although the mechanism studied here destroys the precision offered by the spike, it improves the precision offered exclusively by the curved background.

Figure 4D reveals that the precision of the compass is highly dependent on the rate constants *k*_*f*_ and *k*_*r*_. Hence Fig. 4A–C explore the compass capabilities for biologically reasonable values of these rate constants. The two basic quantities used for characterization of the compass precision are *efficiency* (amplitude) and *directionality* (distribution width) defined as:


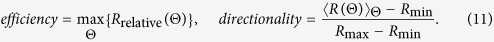


*Efficiency* is simply the maximum value that *R*_relative_ could have, and *directionality* is a measure of the width of the *R*_relative_ distribution. *Directionality* is calculated from 〈*R*(Θ)〉_Θ_, which is the average value of *R*(Θ), while *R*_min_ and *R*_max_ are its minimal and maximal value, respectively. Both quantities are measured in percent, and their values range from 0% to 100%. *Directionality* is defined such that 50% is characteristic for the broadest possible signal, since then 〈*R*(Θ)〉_Θ_ is found exactly in between *R*_min_ and *R*_max_, while 100% corresponds to a very sharp “spike”, at Θ = 0°, leading to 〈*R*(Θ)〉_Θ_ ≈ *R*_max_.

*Efficiency* and *directionality* could be combined into a single unitless measure, which defines *optimality* as:


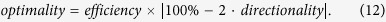


This measure ensures that optimality is 0% for the broadest possible distribution and it is largest for the highest value of efficiency and directionality. The optimality of the model compass is shown in Fig. 4C.

The results shown in Fig. 4A–C puts some constraints on the possible values for the rate constants: *k*_*f*_ cannot be faster than about 1 *μ*s^−1^ in order to preserve some *directionality* (this is consistent with the previously mentioned requirement that *k*_*f*_ ≪ *k*_*S*↔*T*_), and *k*_*r*_ has to be at least two orders of magnitude faster than *k*_*f*_ for any significant *efficiency* and *optimality*, i.e. the regeneration reaction has to be fast. Note that the range of rate constants used in Fig. 4 include the values which are most likely to be found in a realistic biochemical system^28^,^29^. The values that lie outside of this range are, therefore, not discussed.

### Possible biological realization of the chemical compass

The only candidate for a molecular system to host the radical pair-based magnetic compass of migratory birds is cryptochrome, a protein found in the retina of many animals such as migratory songbirds^4^,^41^.

**Figure 6:**
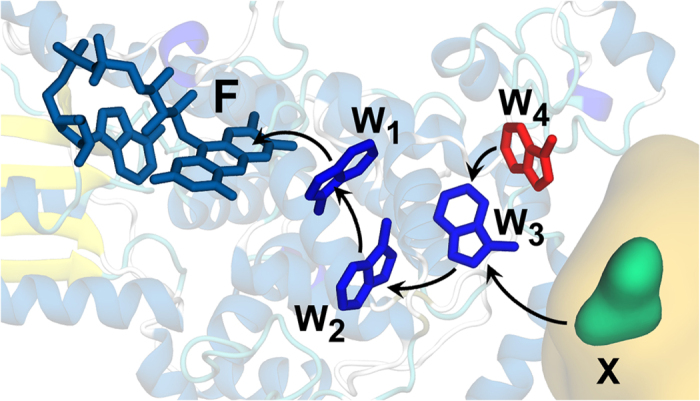
The electron transfer pathway in cryptochrome. The radical pair mechanism introduced in Fig. 2 is possibly hosted by the photoreceptor protein cryptochrome, which is known to operate through a series of electron transfer reactions involving a tryptophan triad (W_1_, W_2_ and W_3_) occuring after photoactivation of the flavin chromophore (F). The putative magnetosensitive radical pair, RP1, is the radical pair involving the chromophore FAD (F) and the third tryptophan in the triad, W_3_. In animal cryptochromes, a fourth tryptophan has been discovered (W_4_), and the radical pair obtained after the 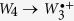 electron transfer process is an alternative candidate for the magnetosensing radical pair, RP1. Both W_3_ and W_4_ are located on the surface of the protein, hence both might be able to interact with external molecules (X) while in their radical state.

Cryptochrome contains the chromophore *Flavin adenine dinucleotide*, FAD, which upon light absorption accepts an electron from a nearby tryptophan amino acid residue, creating a radical pair with the unpaired electrons located on FAD (now FAD^•−^) and the tryptophan residue (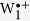). This radical pair is short-lived, as a second and then third tryptophan residue transfer electrons sequentially, leading to a radical pair formation between FAD^•−^ and 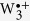 42,43,44,45. This activation mechanism of cryptochrome is depicted in Fig. 6. W_3_ is found at the periphery of the protein, and, therefore, 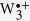 could participate in interactions with external molecular systems. The three tryptophans are conserved in cryptochromes from different organisms^46^,^47^,^48^ and were shown to be important for protein activation. In animal cryptochrome, however, a fourth tryptophan residue has been recently discovered near the third one, and the emergence of a new radical pair, [FAD^•−^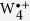], has been observed experimentally^49^. This radical pair with W_4_ is generated very fast by yet another electron transfer, and this has in fact been shown experimentally to happen in less than 20 ns^49^. Whether [FAD^•−^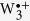] or [FAD^•−^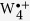] is the magnetosensing radical pair – or whether it may even be a third, yet unknown radical pair – remains to be discovered. It should be noted that the distance between the radicals in the [FAD^•−^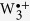] and [FAD^•−^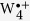] radical pairs is within the range 18–22 Å, which is exactly the range at which the exchange and dipole-dipole interactions are expected to cancel each other^31^, which was one of the assumptions that has been made for the spin chemical model.

No specific interaction partner of the putative magnetosensitive cryptochromes have been confirmed yet, so we do not know whether any radicals or spin-polarized systems may be found near those cryptochromes. However there are many metalloproteins in biological environments, which bind for example iron-sulfur clusters or the iron-containing heme-groups. Such metal containing complexes, and most of the f- and d-block elements in general, usually have unpaired but polarized spins, and would therefore be able to act as the spin polarized electron donors suggested in Fig. S1C. It is, therefore, interesting, that a recently suggested interaction partner for magnetosensitive cryptochrome contains some iron sulfur clusters^50^.

A study using transient absorption spectroscopy has shown, that the radical pair between FAD and W_3_ in garden warbler cryptochrome 1a has a lifetime on the order of milliseconds^12^. This is very interesting, as such long lifetimes (i.e. *k*_*f*_ < 10^−3^ s^−1^) allows for a very precise compass according to Fig. 4C, however, this is without taking spin relaxation into account^51^. It has also been shown that proton transfers to or from either radical in the pair is possible, i.e. deprotonation of W_3_H^•+^ on ns to *μ*s time scales or even faster when nearby histidines are present^52^, or protonation of FAD^•−^ by a nearby aspartic acid^53^. Apparently the aspartic acid required for the latter is only found in plant cryptochromes. Such fast proton transfers as the forward reaction are not favouring the present mechanism – or any other mechanism for that matter – as they would lead to a less sensitive compass.

Another experimental study, performed on *Xenopus laevis* Cry-DASH proteins using TREPR spectroscopy, has concluded that the [FAD^•−^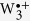] radical pair is formed with a lifetime of at least 6 *μ*s^54^.

It is important to note, however, that the experimental studies mentioned above were performed *in vitro*, hence any interaction partners of cryptochrome that might be present *in vivo* were not considered. Thus similar experiments performed in the presence of possible interaction partners might reveal whether the present mechanism could be realized as in Fig. S1B,C, as that would predict the lifetime of the magnetosensitive radical pair to be much shorter *in vivo* than for isolated cryptochromes.

### Modeling Bird Navigation

Experimental observations of bird flight and ringing recoveries suggest that migratory birds primarily use the magnetic compass to determine the direction of flight^36^. The most detailed information about the flight pattern has been obtained by studying night flyers. According to the observations, the magnetic compass is thought to be recalibrated once per day from celestial cues and is used to establish the flight direction for one night only^36^. The chosen flight direction delivered by the magnetic compass is, however, not precise but rather a random variable around the exact direction, with a typical standard deviation of about 20° 36. Similar distributions has been observed for both caged migratory birds^55^, and for night flyers with attached transmitters^36^. Despite the apparent lack of magnetic compass precision, it is still capable to guide avian navigation quite precisely through continuously repeated readjustment.

**Figure 7:**
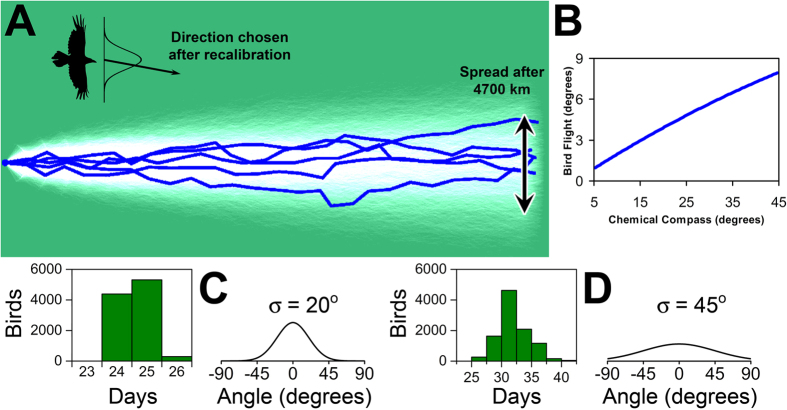
Simulated bird migration. (**A**) The simulated trajectories of 5 birds (blue) navigating solely by the magnetic compass. The birds fly a fixed distance of 200 km each day, and readjust once per day before take-off. Readjustment is simulated by choosing a direction from the probability distribution with a 20° standard deviation (see **C**). In addition to the five highlighted trajectories, 10000 birds were simulated, and their flight trajectories were used to generate a probability distribution of bird dispersion, resulting in the pale trace in the background. (**B**) Repeating the simulations of 10000 birds with different standard deviations of the chemical compass yields different spreads (standard deviations of the positions) of the birds. Here the angular spread of the birds is measured after the travel distance of 4700 km in the migratory direction, as shown in (**A**). (**C**,**D**) The histograms show the number of days needed for the birds to fly 4700 km in the migratory direction, calculated for two different compasses, using the angular probability distributions displayed next to the histograms.

The currently accepted opinion^56^ is that birds fly along straight lines, except for drift due to wind, between readjustments of their magnetic compass. The flight direction is established during readjustment where it is determined by an angular distribution function, provided by the magnetic compass. The conjecture of the present work is that this distribution is analogous to the microscopic distribution, *R*(Θ), of the product rate of a radical pair reaction that depends on the orientation of the bird with respect to the magnetic field, cf. Fig. 7.

The first autumn migration of young birds could be considered as a simple directional flight with an increasing spread, due to the uncertainty of the individual nightly flying sessions. Using the clock-and-compass model, it has been demonstrated that this behaviour results in a parabolic spread of the migratory birds^57^,^58^. It has been observed that adult birds utilize accumulated navigational information from their previous migrations which enables them to correct for displacements and thus improve the flying strategy^59^.

In order to illustrate how the rate of product formation, *R*(Θ), may be interpreted as a direction-dependent probability distribution function, we have simulated the flight of migratory birds. The simulation by itself is not new, as similar simulations has been performed previously^57^, but the important point which we would like to enlighten here is a direct link between the microscopic spin dynamics and the macroscopic navigational capabilities of birds.

Hence we assume that the chemical compass of migratory birds uses *R*(Θ) for navigation, and define a model for studying bird dynamics, governed by this compass. The model employs the following assumptions: (i) the flying birds rely only on the magnetic compass for navigation, while in reality at least the star and the sun compasses are also important^60^,^61^,^62^. These compasses are not included to illustrate the effect of the pure magnetic compass and its precision. (ii) Prior to the flight, each bird recalibrates its compass for a certain time, setting it towards the migration direction. (iii) A random flight direction, governed by the computed direction-dependent probability distribution function, *R*(Θ), is chosen, and a bird is displaced by a fixed distance in that direction. The distance is taken as a characteristic length that a migratory bird travels during one flight. (iv) The birds fly completely independent, i.e. the flight of one bird does not impact the flight of another one.

We have simulated the trajectories of a large number of birds using different compasses, i.e. with different distributions *R*(Θ), to explore the relationship between the precision of the chemical compass and the flight spread of the birds. Figure 7A shows five exemplary simulated trajectories, and a collection of 10000 bird trajectories were used to produce the pale distribution seen in the background. The birds were assumed to fly 200 km per day, and their position was measured once they had flown 4700 km. The angular spread of birds after flying these 4700 km is shown in Fig. 7B, as a function of the microscopic angular spread, i.e. the standard deviation of the compass probability distribution, *R*(Θ). The macroscopic angular spread is almost proportional to the microscopic spread.

Figure 7C,D shows the number of days required to fly 4700 km in the migratory direction using two compasses, i.e. different angular distributions, *R*(Θ), with standard deviations of 20° and 45°, and in fact we see an increase of about 25–30% in the time it takes to make the trip. Hence the need of a precise navigational device is emphasized even more by the flight time than the angular spread of birds. This in turn means that *R*(Θ) must have a narrow distribution, i.e. in terms of Fig. 4 a high optimality value is required.

## Conclusion

Until recently^38^, the study of how birds render a precise chemical compass has received little attention. The present investigation shows how a great improvement of compass precision may be accomplished through a fast spin dependent chemical reaction, provided that this reaction happens in a photoactivated magnetoreceptor molecule and instead of driving the magnetoreceptor molecule into the signaling state, restores its initial inactive state. The notion of a fast spin dependent reaction implies, that this reaction has to be at least an order of magnitude faster than any other reactions occuring after generation of the magnetosensing radical pair, in particular, it should be orders of magnitude faster than the reaction leading to the signaling state of the molecule.

The key assumption of the proposed chemical compass model relies on a fast spin dependent reaction, which is only possible if the host magnetoreceptor molecule is photoexcited continuously multiple times. To justify the feasibility of this repeated activation, we have included an estimate of the time necessary for magnetic compass readjustment under various light conditions in the Supplementary Information. These estimates included an overcast scenario, when migratory birds are known to utilize the magnetic compass^36^. The performed estimates suggest that even in extremely unfavourable light conditions, the readjustment could happen within a few minutes, which support the proposed chemical compass model.

Even though the suggested model shines light on some possibilities for how a precise chemical compass in birds may actually work, the magnetic sense would not be completely understood without a specific description of the biological realization of the compass. So far, much experimental evidence has indicated that the photoreceptor protein cryptochrome could be responsible for this biological realization^1^,^4^,^41^. Cryptochromes are photoreceptor proteins found in the retina of migratory birds – and most other animals as well – and they contain a *flavin adenine dinucleotide* (FAD) cofactor that, upon activation by blue light, generates a radical pair between FAD and a tryptophan located on the surface of cryptochrome. Much is known about the activation mechanism of cryptochrome^1^, but surprisingly little is known about its signaling – in particular how the protein interacts with other proteins in a cell upon activation. Assuming that the chemical compass is realized in cryptochrome, we have explored possible realizations of the crucial fast spin dependent reactions, involving the radical pair between FAD and the tryptophan, and have proposed in the Supplementary Information that this reaction could be rendered (i) through a backreaction, i.e. a reverse electron transfer process that regenerates the initial state of the magnetoreceptor molecule, (ii) an electron transfer reaction facilitated by an external radical or (iii) a reaction with a spin polarized system. Scenario (ii) and (iii) require the involvement of a yet unknown interaction partner, and it, therefore, remain highly speculative whether these possibilities can be realized in cryptochrome. If this is indeed the case, one, however, could expect the radical pair in cryptochrome to have a significantly shorter lifetime *in vivo* than previously measured *in vitro*^12^,^52^.

Migratory birds travel large distances, and due to frequent readjustments of the magnetic compass during the trip, they are able to perform a quite precise flight. Here we linked the microscopic proposition of the chemical compass to the macroscopic scale through the angular probability distribution *R*(Θ), obtained from the spin dynamics of a radical pair. Using a simple model, we have simulated flight trajectories of a large number of birds assuming their navigation to rely on chemical compass distributions, *R*(Θ), of different precision, i.e. for different values of *k*_*r*_ and *k*_*f*_. It was revealed that the precision of the chemical compass has a great impact on not just the spread of birds, but also largely influences the time it takes to make the trip. Hence a precise chemical compass is of great importance, and it should once again be emphasized that a spin chemical mechanism with a fast spin dependent reaction would be able to provide the needed precision.

## Additional Information

**How to cite this article**: Pedersen, J. B. *et al*. Multiscale description of avian migration: from chemical compass to behaviour modeling. *Sci. Rep*. **6**, 36709; doi: 10.1038/srep36709 (2016).

**Publisher’s note:** Springer Nature remains neutral with regard to jurisdictional claims in published maps and institutional affiliations.

## Supplementary Material

Supplementary Information
