# Supplementary material for: Multiscale description of avian migration: from chemical compass to behaviour modeling
Source: Sci Rep. 2016 Nov 10;6:36709. doi: 10.1038/srep36709 (PMC5103213; doi:10.1038/srep36709)
Supplement: Supplementary Information [file srep36709-s1.pdf]

Multiscale description of avian migration: from chemical compass to behavior modeling (Supplementary Information)

J. Boiden Pedersen, Claus Nielsen and Ilia A. Solov'yov*

June 27, 2016

Department of Physics, Chemistry and Pharmacy, University of Southern Denmark, DK-5230 Odense M, Denmark

∗ Email: ilia@sdu.dk

Spin selectivity of the chemical compass

The studied chemical compass model relies on two major assumptions. The first assumption suggests a fast spin-dependent regeneration reaction, and realizations of a fast spin dependent reaction in a biological environment are many. The simplest possibility is the radical pair recombination reaction as illustrated in Fig. S1A, where the electron spins in the magnetic sensing radical pair, RP1, recombine to form an electronically closed shell state in the host magnetoreceptor molecule. This process could happen ultrafast if the radicals are close to each other, e.g. with a rate constant of $\sim 50\ \text{ns}^{-1}$ like the charge recombination reaction in photosystem I from *C. Reinhardtii* (1), and is possible only for the singlet radical pair state, as it is prohibited otherwise due to the selection rules of quantum physics. However the need for the radicals to be close to each other also means, that the exchange and dipole-dipole interactions could be large, and they would generally prevent the mixing of singlet and triplet states, reducing – possibly destroying – the magnetic sensitivity. A fast recombination reaction without such large exchange and dipole-dipole interactions could be acheived if it occurred through a series of intermediate states – i.e. a few subsequent ultrafast short-distance electron transfers. These intermediate states would have increasingly higher exchange interactions, but this would not matter if the singlet-to-triplet mixing has already taken place.

Another credible possibility for a spin selective regeneration reaction could be accomplished with the aid of an external radical, as depicted in Fig. S1B. In this case the spin selectivity arises because the external radical would have an increased chance of reacting with one of the two radicals of the host radical pair, namely the radical of an opposite spin state. Such an external radical could, for example, be hosted in a molecule that is bound to the surface of the host magnetosensor molecule.

A more specific realization of the spin selectivity guided by an exemplary radical could

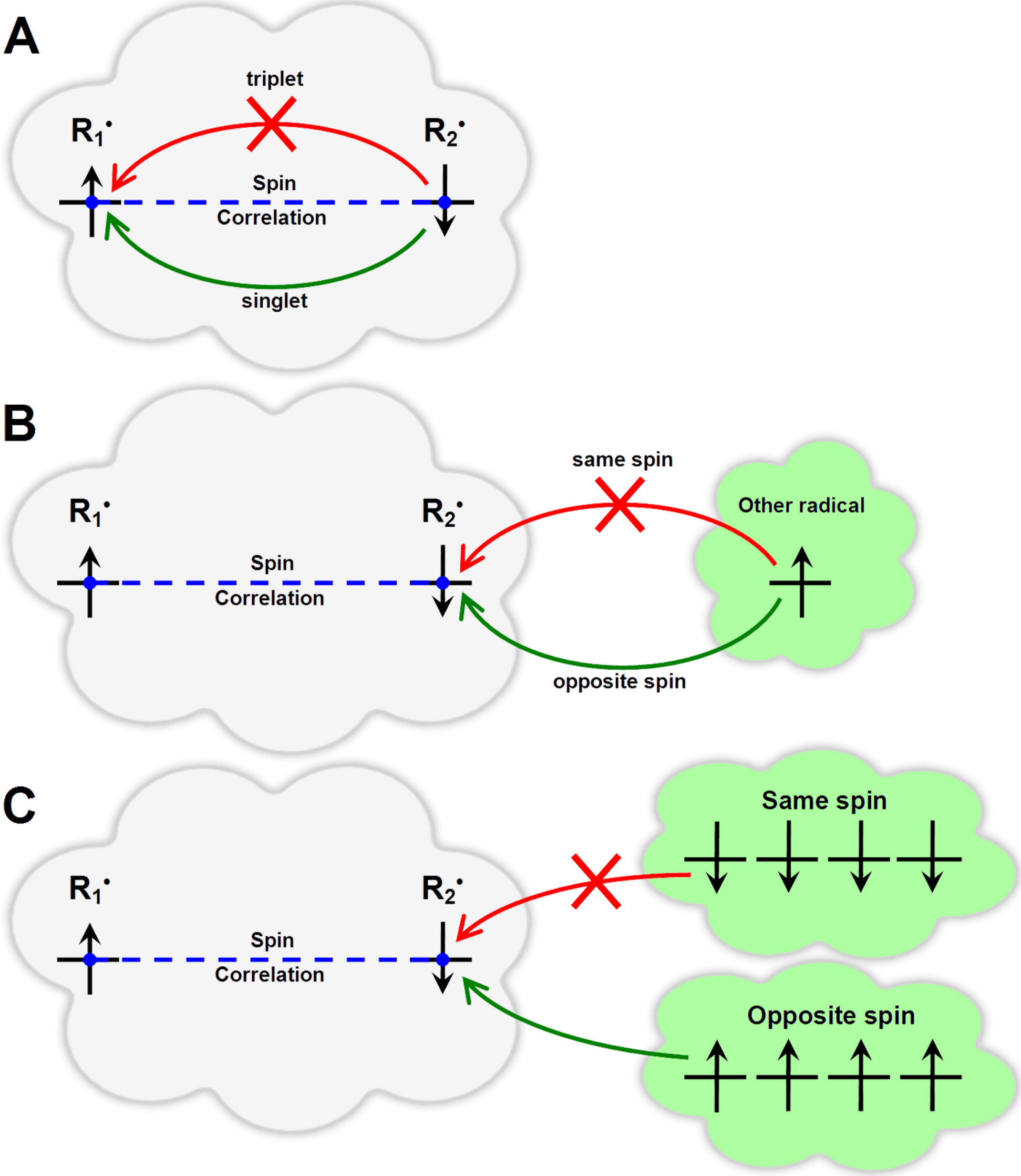

Figure S1: **Possible spin-dependent reactions of the radical pair.** According to the main hypothesis of the model, the primary radical pair, RP1, must be able to undergo a fast, spin dependent quenching process. Some examples of how this spin dependence may arise are illustrated: A fast spin recombination process (**A**) would fulfill the criteria, as only the singlet state of RP1 is allowed to recombine. Alternatively, an external radical (**B**), or a compound with highly polarized electron spins (**C**), would also lead to spin dependent electron transfer reactions, since the radical pair would have an increased chance of reacting with those compounds, which have their spin opposite to that of the radical they are interacting with; the latter being a direct consequence of the Pauli principle in quantum mechanics, and conservation of angular momentum.

also be possibly realized if the magnetoreceptor that hosts the primary RP1 radical pair gets in contact with some spin-polarized particles, e.g. metal atoms or small clusters. In this case spin selectivity is also naturally explained as depicted in Fig. S1C and the corresponding electron transfer could possibly occur ultrafast, depending on the distance between the particles involved.

Cyclicity of the reaction scheme

In constrast to the chemical compass models published earlier (2–6), the present model renders a signal that depends on the amount of photoactivated magnetosensor molecules, and inhomogeneous light conditions would greatly influence the perceived signal. In fact this could be one of the reasons why migratory song birds prefer not to migrate during the day, as continuous sunlight would destroy the magnetic compass sense. On the other hand, this could just as well be a reason that the birds likely recalibrate their magnetic compass once per flight, as the compass would not be very robust during the night either, due to insufficient light. Hence this leaves a “readjustment window”, i.e. a period around sunset where there is neither too much nor too little light available, but just enough to do the magnetic compass adjustment for the night flight. Some birds are, however, known to readjust the magnetic compass during flight, and it has been observed that *Catharus* thrushes oriented in a few minutes after release under extremely dark conditions (7), which corresponded to light intensities between 0.0003 lux and 0.002 lux.

To get a quantitative measure of how much light would be necessary for the present mechanism to work, one needs to estimate the time required to adjust the compass. In order to do this, let us first define the number of photons that enter the eye of a bird per second:

$$\mathcal{N}_{\mathrm{ph}} = \phi A \,, \tag{S1}$$

where ϕ is the photon density (measured in $\frac{\text{photons}}{m^2 \cdot s}$) and A is the cross sectional area of the lens of the bird's eye. Hence, Eq. (S1) defines the number of photons per second that enter the eye, however, not all of those are available to the magnetosensing molecules for a number of reasons: (i) a part of the photons are either reflected by or absorbed in the eye before they even get to the retina, hence χ_r defines the fraction of photons that reach the retina. (ii) Additionally, many other photoreceptors than those responsible for magnetoreception are found in the eye, such that only a fraction of the photons that hit the retina are actually absorbed by the magnetosensing molecules; the latter fraction is thus denoted as χ_f. The amount of photons per second available for the magnetosensing molecules, therefore, reads:

$$\mathcal{N}_{\text{avail}} = \chi_r \chi_f \mathcal{N}_{\text{ph}} \,. \tag{S2}$$

Next we need to establish the number of excitations of the magnetosensing molecules required by the proposed spin chemical compass to function properly. Let λ be the number of consecutive excitations required for a single magnetoreceptor molecule to end up in the signaling state, and ζ be the minimal number of magnetoreceptor molecules in the retina that are required to be in the signaling state in order for a bird to utilize the magnetic compass. The required number of photons could thus be calculated as:

$$N_{\text{req}} = \lambda \zeta \,. \tag{S3}$$

Hence the signaling time, i.e. the time for the bird to perceive the magnetic field, is:

$$\tau = \frac{N_{\text{req}}}{2\mathcal{N}_{\text{avail}}} = \frac{\lambda \zeta}{2\chi_r \chi_f \phi A} \,, \tag{S4}$$

where the factor of 2 takes into account that the bird has two eyes, both capable of perceiving the magnetic field (8). One should note that Eq. (S4) is independent of $R(\Theta)$ and, therefore,

independent of the magnetic field effect.

Assuming a spin dependent reaction with $k_r = 10\ \text{ns}^{-1}$ and a forward reaction occuring with a rate of $k_f = 0.1\,\mu\text{s}^{-1}$, one derives $\lambda \approx k_r/k_f = 10\ \text{ns}^{-1}/0.1\,\mu\text{s}^{-1} = 10^5$, being the number of excitations per molecule needed to reach one signaling state. There are no clear indications about the photoreceptor fraction χ_f at present, thus we put $\chi_f = 25\%$, i.e. assuming that $^1/_4$ of the photons that hit the retina are utilized to excite the magnetosensing molecules. Also, we assume that half the photons flying towards the eye is either reflected or absorbed before it even reaches the retina, hence $\chi_r = 50\%$. The lens of a european robin's eye has a radius of about 1 mm, hence $A = \pi r^2 \approx 3\ \text{mm}^2$. For light of 380-720 nm wavelength, the relationship between the photon density, ϕ, and the light intensity, I, is approximately $\phi = 1.2I \cdot 10^{16} \frac{\text{photons}}{\text{s}\cdot\text{m}^2\cdot\text{lux}}$ (9), which finally allows to express the signaling time as:

$$\tau = \zeta \frac{10^5\,\text{photons}}{2 \cdot 0.5 \cdot 0.25 \cdot 1.2I \cdot 10^{16}\,\frac{\text{photons}}{\text{s}\cdot\text{m}^2\cdot\text{lux}} \cdot 3\,\text{mm}^2} = \zeta\,\frac{10^{-4}}{9I}\,\text{s}\cdot\text{lux}\,. \tag{S5}$$

This estimate could be simplified further, once the minimal number of activated magnetosensor molecules in the retina is known. It has been shown for other photoreceptors, e.g. rhodopsins (10), that a single photon is sufficient to trigger a nervous signal with a membrane potential of about 1 mV, and one could speculate that this may also be the case here. Alternatively, many thousands of excited magnetosensor molecules may be required to initiate a specific response. For the sake of an estimate, we, therefore, chose a generic value $\zeta = 1000$. This value is motivated by the fact that a single magnetosensor molecule in the signaling state might not be enough, and would surely not be robust enough to function as a chemical compass. Following this assumption for ζ, one obtains:

$$\tau = \frac{0.1}{9I}\,\text{s}\cdot\text{lux}\,. \tag{S6}$$

Let us now consider three illumination scenarios: sunset/sunrise, night with a full moon, and

an overcast moonless night. Typical light intensities in these three cases are 10 lux, 0.01 lux and 0.001 lux, respectively (9), and according to Eq. (S6) lead to signaling times of about 1 ms, 1 s and 10 s, respectively. Since these are just the signaling times, one must assume that the actual compass readjustment time is longer, i.e. the bird needs to collect geomagnetic information from different orientations during a headscan. The signaling times are still quite reasonable, however, since even for the very dark scenario the compas readjustment could be accomplished within a minute or two – depending on the exact readjustment mechanism, which is presently unknown. It is, however, important to stress that due to the uncertainty regarding some of the parameters the signaling could happen on a different time scale and the present estimates are used to illustrate that even extremely dark light conditions are seemingly sufficient to utilize the proposed compass. The estimates are, however, still consistent with the experimental evidence (7) that *Catharus* thrushes could reorient within a few minutes in almost extreme darkness.

References

[1] Holzwarth, A. R., Mu1ller, M. G., Niklas, J. & Lubitz, W. Charge recombination fluorescence in photosystem i reaction centers from chlamydomonas reinhardtii. *Journal of Physical Chemistry B* **109**, 5903–5911 (2005).

[2] Ritz, T., Adem, S. & Schulten, K. A model for photoreceptor-based magnetoreception in birds. *Biophysical Journal* **78**, 707–718 (2000).

[3] Maeda, K. *et al.* Magnetically sensitive light-induced reactions in cryptochrome are consistent with its proposed role as a magnetoreceptor. *Proceedings of the National Academy of Sciences, USA* **109**, 4774–4779 (2012).

[4] Rodgers, C. & Hore, P. Chemical magnetoreception in birds: the radical pair mechanism. *Proceedings of the National Academy of Sciences, USA* **106**, 353–360 (2009).

[5] Solov'yov, I. A. & Schulten, K. Reaction kinetics and mechanism of magnetic field effects in cryptochrome. *The Journal of Physical Chemistry B* **116**, 1089 – 1099 (2012).

[6] Solov'yov, I. A., Chandler, D. E. & Schulten, K. Magnetic field effects in *arabidopsis thaliana* cryptochrome-1. *Biophysical Journal* **92**, 2711 – 2726 (2007).

[7] Cochran, W., Mouritsen, H. & Wikelski, M. Migrating songbirds recalibrate their magnetic compass daily from twilight cues. *Science* **304**, 405–408 (2004).

[8] Hein, C. M., Engels, S., Kishkinev, D. & Mouritsen, H. Robins have a magnetic compass in both eyes. *Nature* **471**, E11–E12 (2011).

[9] Solov'yov, I., Mouritsen, H. & Schulten, K. Acuity of a cryptochrome and vision-based magnetoreception system in birds. *Biophysical Journal* **99**, 40–49 (2010).

[10] Nelson, D. L., Cox, M. M. & Lehninger, A. L. *Lehninger principles of biochemistry* (W.H. Freeman and Company, New York, 2013), 6th edn.
